# Increased Asymmetric Perfusion of the Cerebral Cortices and Thalamus Indicates Individuals at Risk for Bipolar Disorder: A Family Cohort Single Photon Emission Computed Tomography Neuroimaging Study

**DOI:** 10.3389/fpsyt.2022.829561

**Published:** 2022-05-10

**Authors:** Mary McLean, Theodore A. Henderson, Dan G. Pavel, Phil Cohen

**Affiliations:** ^1^Private Practice, Toronto, ON, Canada; ^2^The International Society of Applied Neuroimaging (ISAN), Denver, CO, United States; ^3^The Synaptic Space, Inc., Denver, CO, United States; ^4^Neuro-Luminance, Inc., Denver, CO, United States; ^5^Dr. Theodore Henderson, Inc., Denver, CO, United States; ^6^The Neuro-Laser Foundation, Denver, CO, United States; ^7^PathFinder Brain SPECT, Deerfield, IL, United States; ^8^Lions Gate Hospital, Vancouver, BC, Canada; ^9^Department of Radiology, University of British Columbia, Vancouver, BC, Canada

**Keywords:** biomarker, thalamus, psychiatry, perfusion, prodromal, seizure, single photon emission computed tomography, ADHD

## Abstract

Bipolar disorder is a significant mental illness affecting over 4 million people in North America and approximately 46 million worldwide. While the onset of bipolar disorder is typically in late adolescence and early adulthood, the correct diagnosis can be delayed for several years. This delay can result in inappropriate pharmaceutical interventions, loss of career or productivity, suicide, family hardship, and unnecessary expense. Moreover, prolonged untreated or inappropriately treated bipolar disorder may cause damage to the brain. Early diagnosis is a critical need to circumvent the damage, suffering, and expense caused by the current delay. Brain perfusion single photon emission computed tomography (SPECT) neuroimaging reveals visual correlates of brain function. Herein, a family cohort all with bipolar disorder is described and their symptoms correlated with findings on the individual SPECT brain scans. The family consisted of two parents and three children (one female). The scans were interpreted by a panel of experts. Then a *post hoc* region-of-interest (ROI) analysis was conducted on SPECT data normalized to the cerebellum maximum with comparison to similarly normalized data from a normative sample. These findings support two distinct patterns of SPECT perfusion scan changes that can be found in individuals with bipolar disorder. In addition, these findings indicate that SPECT scan findings may be predictive of individual risk for progressing to symptomatic bipolar disorder. While preliminary, the findings in this cohort support the need for larger, diverse cohort studies of bipolar and control subjects to assess the predictive value of these particular SPECT perfusion findings in bipolar disorder.

## Introduction

Bipolar disorder is a spectrum of mood disorders with significant morbidity. Bipolar I disorder, previously known as manic depressive disorder, is characterized by one or more manic episodes, alternating with episodes of depression or euthymia. Bipolar II disorder is characterized by cyclic episodes of hypomania alternating with episodes of depression or euthymia. A third presentation, referred to as a “Mixed State,” features negative feelings of depression along with agitation, restlessness, or a “wired” state of mania. Besides these three defined entities, the spectrum of mood disorders with a cyclic component is broad and diverse. Bipolar disorder has a prevalence of 1%, which equates to approximately 46 million patients worldwide (1). In North America, the prevalence is approximately 1–1.5% (2) or approximately 4 million people.

While the onset of bipolar disorder is typically in late adolescence and early adulthood, the correct diagnosis can be delayed for several years. Retrospective studies in multiple nations have shown delays of greater than 5 years between the onset of symptoms and the correct diagnosis (3–6). However, some studies have shown delays of greater than 10 years (6–8). In addition, patients are often initially treated with antidepressants or stimulants (5, 9–11), which can lead to exacerbation of symptoms, manic episodes, and a worsening of the course of the illness. For example, antidepressant monotherapy is associated with increased incidence of manic episodes and suicide (12, 13). This diagnostic delay can take on more serious consequences in the case of early-onset bipolar disorder among children. Treatment with stimulant medications for presumptive attention-deficit/hyperactivity disorder (ADHD) can lead to mania, hypomania, or accelerated cycling (12, 14–18). Alternative treatments for ADHD, such as atomoxetine, have also been shown to precipitate mania in children (19, 20). Early diagnosis and differentiating bipolar disorder from ADHD are key steps in reducing later morbidity.

Bipolar disorder is the 12th leading cause of disability worldwide (21). Untreated bipolar disorder leads to impaired ability to work, difficulty maintaining relationships, and academic failure (7, 22). Being untreated or incorrectly treated (due to misdiagnosis with unipolar depression) amplifies the already heavy burdens associated with bipolar disorder. Despite treatment, patients with bipolar disorder have higher healthcare costs, more frequent emergency room visits, more frequent hospitalizations, and more frequent psychiatric appointments (23, 24). Despite treatment, patients with bipolar disorder also experience higher rates of unemployment (25) lower levels of work productivity (26), and higher rates of short-term disability (24). Patients with bipolar disorder are more likely to have comorbid medical problems and a shorter lifespan (27, 28). Furthermore, patients with bipolar disorder are more likely to fail to adhere or comply with a medication regimen long-term. Multiple factors underlie this tendency toward non-adherence, including impulsivity, personality traits, anxiety, substance use, beliefs, and impaired insight (29). Adherence issues created added costs for both patients and providers/systems (29). The individual medical expenses associated with bipolar disorder are estimated to range from $11,000–$46,800 (USD) annually (24). The direct and indirect costs of bipolar disorder have been estimated at between $194–$219 billion (USD) annually in the United States (23, 27).

Altogether, the diagnostic delay can result in inappropriate pharmaceutical interventions, loss of career or productivity, family hardship, suicide, and unnecessary expense. Moreover, prolonged untreated or inappropriately treated bipolar disorder may cause damage to the brain (30). Early diagnosis is a critical need to circumvent the damage, suffering, and expense caused by the current delay in diagnosis and treatment.

### Can Neuroimaging Provide a Biomarker?

Given the substantial overlap of symptoms between bipolar disorder, unipolar depression, ADHD, and oppositional defiant disorder (ODD), a biomarker for bipolar disorder would be invaluable. Short of a definitive molecular biomarker, neuroimaging findings which are consistent and predictive would aid the diagnostic process. It is important to understand that the predictive value of a neuroimaging finding need not be perfect. A marker with reasonably high sensitivity and specificity (>75%) would be sufficient to warrant pharmacological interventions targeting a bipolar phenotype. Since the medications to treat bipolar disorder include several with very low risks and side effects, the danger of making an incorrect pharmacological choice also is low. On the other hand, in the case of localizing a seizure focus for surgical ablation, high sensitivity and specificity would be paramount. Nevertheless, positron emission tomography (PET) for localizing seizure foci has a sensitivity of 65–95% and a specificity of 90–95% (31, 32). Hence, it is *not unreasonable* to explore neuroimaging markers for bipolar disorder that would expedite reaching the correct diagnosis and treatment even if they lack perfect 100% sensitivity and specificity. Certain findings that we have consistently observed in our clinical practices utilizing single photon emission computed tomography (SPECT) neuroimaging in the evaluation of literally thousands of patients with bipolar disorder also have been described in the research literature.

### Perfusion SPECT Findings in Bipolar Disorder

SPECT neuroimaging has demonstrated moderate consistency across multiple studies in both depressed and manic states. Early perfusion SPECT studies utilizing ^133^Xenon gas focused only on cerebral cortex (33–37) and found decreased frontal lobe perfusion during bipolar and unipolar depression. With the advent of stabilized tracers, such as *99^m^* Tc-ethyl cysteinate dimer (ECD) and *99^m^* Tc-hexamethylpropyleneamine oxime (HMPAO), additional observations became evident. Decreased frontal lobe perfusion is widely reported in bipolar depression (38–43). In contrast, mania can present with more profound hypoperfusion in the orbitofrontal cortex and the anterior temporal lobes (42, 44). Moreover, a left-right asymmetry has been reported by some (36, 45, 46). This asymmetry also can be found in the thalami (42, 47–49). For example, Juckel et al. (50) described a case of bipolar disorder with ultrarapid cycling that received a SPECT scan during a manic phase and again during a depressive phase (within 48 hours). During the manic phase, thalamic perfusion was markedly elevated and asymmetrical. On the following day, when the patient was in a depressed phase, thalamic perfusion was less asymmetrical (50).

Lithium withdrawal frequently precipitates mania and was used to elucidate mood and perfusion changes in a sample of 7 patients converted from a euthymic state (on lithium for over one year) to a manic state. Perfusion increased in the posterior temporal and parietal cortices (51). Recently, perfusion SPECT scans were examined in a group of patients with bipolar I during a manic episode and 6 months later (52). A sample of 10 patients with bipolar mania (diagnosed based on DSM-IV criteria and an elevated Young Mania Rating Scale score) underwent perfusion SPECT with statistical comparison to a normative database. Overall, perfusion was increased more in the right hemisphere than in the left hemisphere. Compared to the normative database, perfusion was elevated throughout the cerebral cortices, including the frontal lobes. The perfusion in the temporal poles bilaterally stood out as markedly elevated. At 6-month follow-up, the patients were all euthymic. Perfusion was still elevated in the bilateral temporal and parietal cortices, but the bilateral frontal cortices showed lower perfusion than the normative database. Notably, the asymmetry still was present (52). Our extensive clinical experience spanning decades and tens of thousands of SPECT scans support the consistent finding of asymmetry in the cortex and, prominently, in the thalamus (48).

### Other Neuroimaging Modality Findings in Bipolar Disorder

Other neuroimaging modalities provide supporting evidence. PET neuroimaging using ^18^F-fluorodeoxyglucose (FDG) to visualize regional glucose uptake as a measure of regional brain activity have shown the thalamus to be involved in mood disorders. Milak et al. (53) examined FDG PET scans of 298 medication-free patients with depression. They performed factor analysis to correlate brain activity with various factors of the Hamilton Depression Rating Scale. Increased metabolism in the thalamus, as well as the subgenual anterior cingulate, subgenual basal forebrain, posterior cingulate, and ventral striatum correlated positively with the emotional metrics of depressed mood, guilt, suicidal ideation, helplessness and hopelessness. In contrast, psychotic symptoms endorsed on the Hamilton Scale did not correlate positively or negatively with any brain region, while the symptoms of low motivation correlated with the dorsolateral prefrontal cortex, but not with the thalamus. Brody et al. examined metabolism changes in 24 patients at baseline and after treatment for depression (54). When compared to a control cohort of 16 subjects, patients with depression showed higher metabolism in the bilateral thalamus, as well as the prefrontal cortex and striatum. They also noted that decreases in the Hamilton Depression Scale score were correlated with decreases in thalamic metabolism. Ketter et al. (55) examined treatment-resistant, rapid-cycling bipolar disorder patients compared to age-matched healthy controls using FDG-PET. They found that bipolar disorder was characterized by increased metabolism in the thalamus, striatum, and right amygdala, but decreased metabolism in the frontal cortex (55). Also, they noted an increase in cerebellar metabolism, which appeared to be independent of mood state.

PET perfusion studies of bipolar mania using ^15^O-H_2_O also found hypoperfusion of the orbitofrontal cortex (56) but increased anterior cingulate perfusion (57). Similarly, fMRI studies show increased perfusion in multiple cortical areas and the thalamus, in combination with decreased ventral frontal cortex perfusion (most often more severe on the right), has been reported in unmedicated bipolar disorder patients while performing a motor task (58). Similar fMRI findings have been reported in a number of studies, as well as phase-dependent changes, such as increased thalamic perfusion (greater on the left) during mania and decreased asymmetric perfusion of the ventral frontal cortex (59). Altogether, these findings are consistent with an asymmetrical dysfunction within prefrontal-striatal-thalamic and amygdala-thalamic-cortical circuits, as well as the ventral striatum-limbic-thalamic circuit, which are believed to underlie the pathophysiology of bipolar disorder (59, 60).

### A Clinical Pearl

In the present study, a diverse group with extensive experience reading and interpreting SPECT scans focused attention on a retrospective review of a single family in which every member manifested mood disorder symptoms. The SPECT neuroimaging findings in each member of this family demonstrated findings we have typically seen and associated with bipolar disorder in our diverse and international practices – namely, increased asymmetrical perfusion of the thalamus and increased asymmetrical perfusion of the cerebral cortices. This unique nuclear family – all with the diagnosis of bipolar disorder and providing perfusion SPECT scan data – is a first inroad into defining a working hypothesis about potential neuroimaging biomarkers for bipolar disorder.

In addition, these findings are presented herein using a novel display methodology and subjected to quantitative analysis using a novel method, both of which are described in detail in the methods, due to their innovation.

## Materials and Methods

The method of analysis of the SPECT scans for interpretation and the search for similarities was by expert consensus panel. The scans were presented to a group of clinicians from diverse backgrounds that all had extensive experience with the interpretation of SPECT scans. The group included four nuclear medicine physicians, one general psychiatrist, one child/adult psychiatrist, one psychiatrist cross-trained in nuclear medicine, and one general practitioner with advanced training in psychiatry. The physicians practice in either the United States, Australia, or Canada. Collectively the committee has read/interpreted/analyzed over 61,700 perfusion SPECT scans. Specifically, they are: Dan Pavel, MD (over 20,000 scans); John Michael Uszler, MD, MS (over 10,000 scans); Theodore Henderson, MD, PhD (over 20,000 scans – over 14,000 scans in published articles); Phil Cohen, MD, FRCPC (over 3,000 scans); J. Cardaci, MBBS, FAANMS, FRACP (over 4,000 scans); Yin-Hui Siow, MD, FRCPC (over 3,000 scans); John F. Rossiter-Thornton, MB, FRCPC (over 800 scans); Mary McLean, MB, ChB, FRCP (over 600 scans); Muriel J. van Lierop, MBBS, MDPAC(M) (over 300 scans) (61–65).

The patients were all members of a single family in the clinical practice of one of the authors (MML). The SPECT scans were performed as part of the ongoing assessment and treatment of these patients, each of whom was diagnosed with bipolar disorder. As such, the symptom data collected on these patients was not quantitative, nor systematic. Rather, it was clinical records of the assessment and management of these patients. The study is retrospective and naturalistic in that regard. However, the analysis of the scans utilized modern displays and interpretation by a group of clinicians with the experience of collectively interpreting thousands of SPECT scans. A subsequent *post hoc* regions of interest (ROI) analysis was performed comparing the results of the family cohort to a group of non-aged-matched patients who did not have the diagnosis of bipolar disorder.

Each family member’s case was presented to the group *via* monthly electronic conferences. A detailed history of the patient was provided verbally without identifying information. Then the de-identified SPECT scan results of that patient were shown and discussed by the group. The SPECT images were presented first in tomograms (horizontal, coronal, and sagittal) displayed in a polychromic color scale based on normal physiological cerebral perfusion. Then the scan results were shown in 3-dimensional displays wherein cortical perfusion falling below 60% of cerebral maximal perfusion presents as holes or depressions. Lastly, the results were displayed as a 3-dimensional model using the polychromic color scale. During the discussion, all displays were accessed and deliberated upon until a consensus opinion was reached.

### Scanning Protocol

The scans themselves were performed at Mt. Sinai Hospital, Toronto, ON. The nuclear medicine department followed standard imaging procedures. The patient was positioned under dimmed lights lying supine. An intravenous line was started. The HMPAO was administered after allowing the patient to rest quietly for at least 5 minutes. After a 30-minute washout period, the patient was placed in a Picker Prism 3000 three-head gamma camera equipped with a low-energy, ultra-high resolution fan beam collimator. A step and shoot method of acquisition was used with 120 steps, 3 degrees per step, using 128 × 128 matrix and continuous acquisition. Approximately 22–30 seconds per stop yielded 3–4 million counts for each scan. The data was then passed through a ramp backprojection filter and a 3D Butterworth filter (order 5.0, cutoff frequency 0.2–0.3 cycle per pixel). Non-neural structures were masked. Attenuation correction was applied by the method of Chang (66, 67).

### Analysis and Display Protocols

**FIGURE 1:**
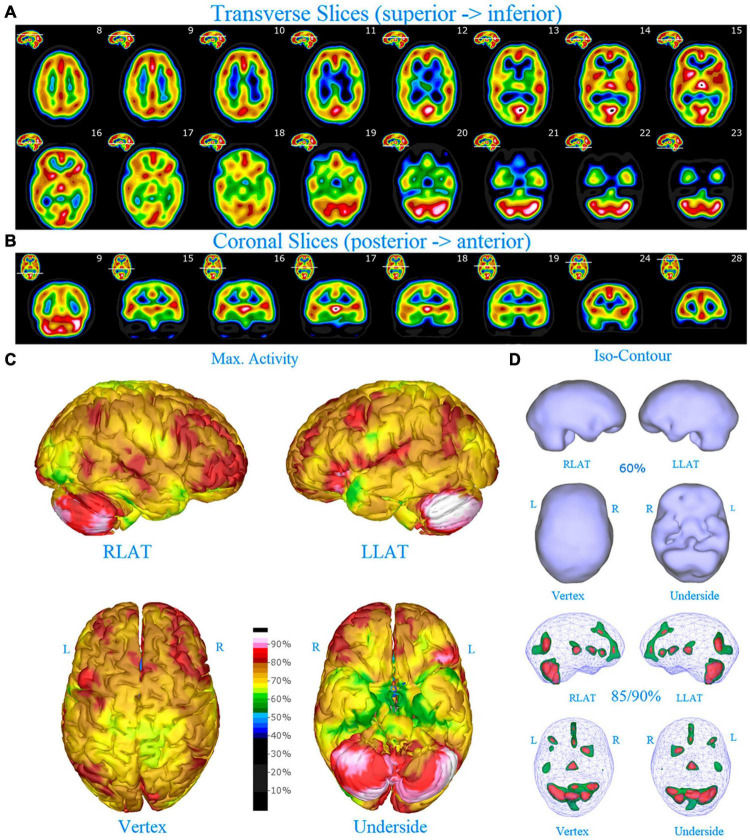
Perfusion SPECT scan of normal control. **(A)** Horizontal tomograms from a *99^m^* Tc-HMPAO perfusion SPECT scan spanning much of the brain from near superior surface to lower cerebellum (see locator images). **(B)** Coronal tomograms spanning from anterior margin of cerebellum to anterior cingulate (see locator images). The color scale is scaled relative to the patient’s mean cerebral perfusion. Mean blood flow (72%) is in yellow. Color shifts occur at approximately every 0.5 SD (3%) relative to the patient’s mean. Details of the brain can be appreciated, including the thalamus, head of the caudate nuclei, lentiform nuclei, anterior cingulate gyri, and distinct cortical regions. The perfusion of the thalamus (white with black central dot), right caudate (on the left side of the tomogram), visual cortex and cerebellum are highest in this example. **(C)** The modern SPECT scan displayed in 3-dimensional reconstruction. Right lateral (RLAT), left lateral (LLAT), Vertex, and underside views are provided. The color scale is the same as panel **(B)**. **(D)** Isocontour representations of the SPECT data in 3 dimensions. Surface isocontour representations allow visualization of areas of decreased cortical perfusion. Areas in which perfusion falls below 60% of the maximal cerebral blood flow appear as depressions or holes depending on how far below 60% the perfusion falls. Wireframe representations allow visualization of areas of increased perfusion. Areas with perfusion at 85% of the maximal cerebral blood flow appear in green, while areas at 90% of maximal cerebral blood flow appear in red. It is evident that the thalamus, right caudate, cerebellum, and visual cortex are perfused at 90% of maximal cerebral blood flow. This corresponds to what is seen in the tomograms.

The data was then processed using a proprietary method (Good Lion Imaging, Columbia, MD, United States). Briefly, this methodology visualizes the reconstructed data of a brain SPECT scan in three distinct but complementary displays. Extra-cerebral activity is masked from the volumetric data and the data is scaled to the maximum value in the brain. Each display is designed to assist the reader in easily extracting the key perfusion information necessary to arrive at a consistent and meaningful interpretation. The display has the following features: (1) The standard set of 2-D orthogonal slices (horizontal, coronal, sagittal) are displayed in a unique color scale with 21 discrete, equal width, color steps in a geographic progression which allows for a semi-quantitative evaluation. The color scale includes a threshold setting at 40% of maximal perfusion within the brain (usually cerebellum) to suppress background activity which otherwise would distract from the usable information. This threshold setting is the floor of the first color step. A 4th slice orientation along the temporal lobe axis is provided to visualize the distribution within this lobe in a more natural way. (2) The so-called multi-volume threshold display is a 3-D presentation of the volumetric data in 6 orientations using four different iso-contour thresholds. The lower two thresholds of 55% and 65% of maximal brain perfusion, respectively, help in identifying areas of hypo-perfusion as well as their extent. The upper two thresholds of 85% and 90% of maximal brain perfusion, respectively, help to identify possible areas of hyper-perfusion. (3) A set of 3-D surface projections mapping the maximum cortical activity on a surface rendering of the brain. This display is somewhat analogous to the polar plot in cardiac imaging, providing an overview of brain activity distribution in a single view. These 3-D displays have the advantage of illustrating both subtle widespread cortical abnormalities, as well as illustrating general patterns of both hypoperfusion, but, in particular, increased perfusion, in a manner more easily appreciated compared to tomograms. The latter two displays, while presented in a static form, can also be made available in a dynamic, rotating cine format, to enhance the 3-D impression which are extremely useful in communicating the imaging results to the referring clinicians. See Figure 1 for illustrative example.

### *Post hoc* Regions of Interest Analysis

A *post hoc* ROI analysis was conducted to provide a secondary confirmation of the expert consensus visual interpretations. A total of 67 ROIs were uniquely developed based on atlas normalized SPECT and MRI datasets. This set of region templates was then applied to each scan after Talairach transformation using the Neurostat software (68) and normalization to the cerebellar maximum. Median and maximum counts for each region were determined. The data were presented in bar graph form to visualize the regional activity on a scale wherein the cerebellar maximum equaled 100% for each patient for each scan. Then, the median count value of a normative sample for each ROI was indicated by a white horizontal line on the bar. Similarly, the maximal count value of a normative sample for each ROI was indicated by a red horizontal line on the bar. This allowed the simultaneous comparison of the counts in each ROI relative to the patient’s own cerebellar maximum counts and to the median and maximum of the normative sample. The intent of this counts-based ROI analysis was to perform a semi-quantitative *post hoc* recheck of the committee’s findings which were derived from visual reading of the scans, not as a stand-alone analysis.

An abbreviated history and key scan findings will be presented here for each patient in the family case series, followed by extrapolated results on the SPECT neuroimaging findings in bipolar disorder.

## Results

The family consisted of five members. The father (Patient A) was scanned in 2009 at age 47. The mother (Patient B) was scanned in 2012 at age 48. The oldest daughter (Patient C) was scanned in 2009 at age 10 and again at age 15 (2014). The older son (Patient D) was scanned in 2012 at age 10 and again at age 13. The younger son (Patient E) was scanned (in 2012) at age 6 and again at age 9. Each patient’s case history and scan analysis will be presented in turn. A SPECT scan from a patient without psychiatric conditions is shown in Figure 1 for comparison purposes.

### Patient A

Patient A (the father) grew up in rural Ontario with a mother who was housebound and a father with alcoholism. He displayed numerous signs of mania and hypomania as a child. He leaped off a cliff when he was 12 years old, engaged in heavy drinking and glue-sniffing, and arrived drunk at a school function and danced about the gymnasium with a teacher’s wife at age 14. He left home upon high school graduation and was estranged from his father. He struggled with depression and suicidal ideation in college.

When he first presented for treatment, Patient A was 38 years old. He had participated in psychotherapy for several years and had started Paroxetine one year prior. His mood had worsened on Paroxetine and it was discontinued. Nevertheless, depression and anxiety escalated, and notably, he developed verbal aggression, which was out of character. The patient had an inconsistent work history, characterized by periods of intense activity alternating with periods of listlessness, procrastination and anxiety. The patient commented, “I groove on adrenaline and anger.” Suspecting a bipolar mixed state, the clinician started Moclobemide (reversible MAO-I). The patient experienced a significant reduction in aggression and irritability, as well as reduced alcohol use and risky behavior. After one year, Moclobemide was discontinued. The patient experienced a renewed depression, as well as increased anxiety. He was restarted on Moclobemide. He was stable for two years and then the medication lost efficacy. The patient was switched to Bupropion. Later, Adderall was added due to difficulties with concentration.

**FIGURE 2:**
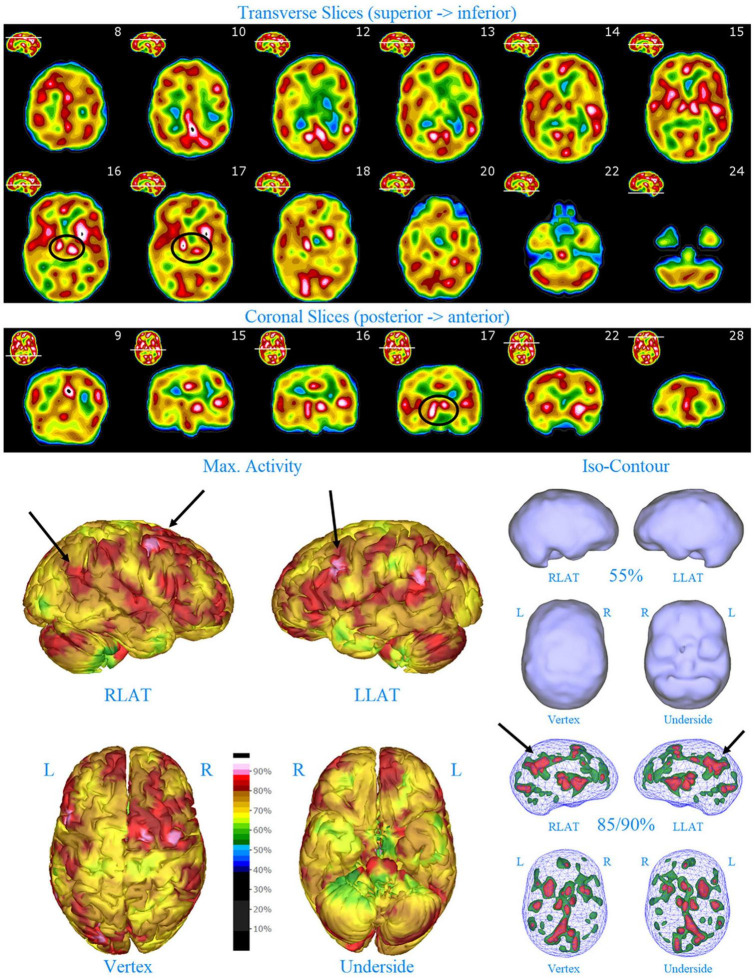
Perfusion SPECT scan of Patient A. Arrangement, color scales, and views are identical to Figure 1. Perfusion of the thalamus is increased and asymmetrical (circled in horizontal and coronal tomograms). Perfusion also is increased in the basal ganglia, particularly on the left (above and to the right of the encircled thalamus). Multiple areas of increased perfusion are seen throughout the cerebral cortices in both the 3-dimensional reconstruction and the wireframe isocontour representations (arrows). The frontal lobe on the right show more substantial increases in cerebral perfusion.

After some years of stability on Buproprion and Adderall, Patient A’s mood crashed, accompanied by overwhelming panic. He was described by his wife as “a 3-year-old in a 47-year-old body.” His symptoms included depression, agitation, severe anxiety, panic attacks, and irritability. A SPECT Scan was obtained (Figure 2) and, based on the results of the scan, psychiatric stability was achieved by adding Gabapentin to his regimen. In sessions, for the first time in many years, the couple was able to comfort each other.

At various times, Patient A had brief trials of Oxcarbazepine and Lurasidone, while in a mixed state. Neither was successful. Overall, the patient did best on the regimen of Adderall, Bupropion, and Gabapentin, which were determined based on the SPECT scan results.

Single photon emission computed tomography scan findings for Patient A (Figure 2) included: multiple cortical areas of increased perfusion (“hot spots”), increased frontal lobe perfusion (right > left), dramatically increased perfusion of the posterior cingulate gyrus, and increased perfusion of the basal ganglia (left > right). Notably, the perfusion of the thalamus was asymmetrical in its perfusion.

### Patient B

Patient B (the mother) was initially seen in psychotherapy following the breakup of her first marriage at age 30. She is described as “a caregiver to all” and is called on by members of her family in any moment of crisis. She is a peacemaker. She has always worked excessively and in the not-for-profit sector, in areas of community development. She is described as being very organized, busy, and a tower of strength.

The Patient B began taking Adderall at age 36, when she identified similar struggles with concentration as described by Patient A. She initially became depressed during her third pregnancy coinciding with a period of financial crisis. She stayed in bed for 2 weeks after the birth. Two years later, she again expressed concerns about her ability to concentrate and Adderall was again started. She continued to work prodigious hours and attended graduate school (on a full scholarship) when the youngest child was 6 years old. During this time, she would be in her office 10–12 hours per day, 7 days per week. Patient B described herself as “Putting a quart into a pint pot” and “I’m too busy to see the whole picture.”

**FIGURE 3:**
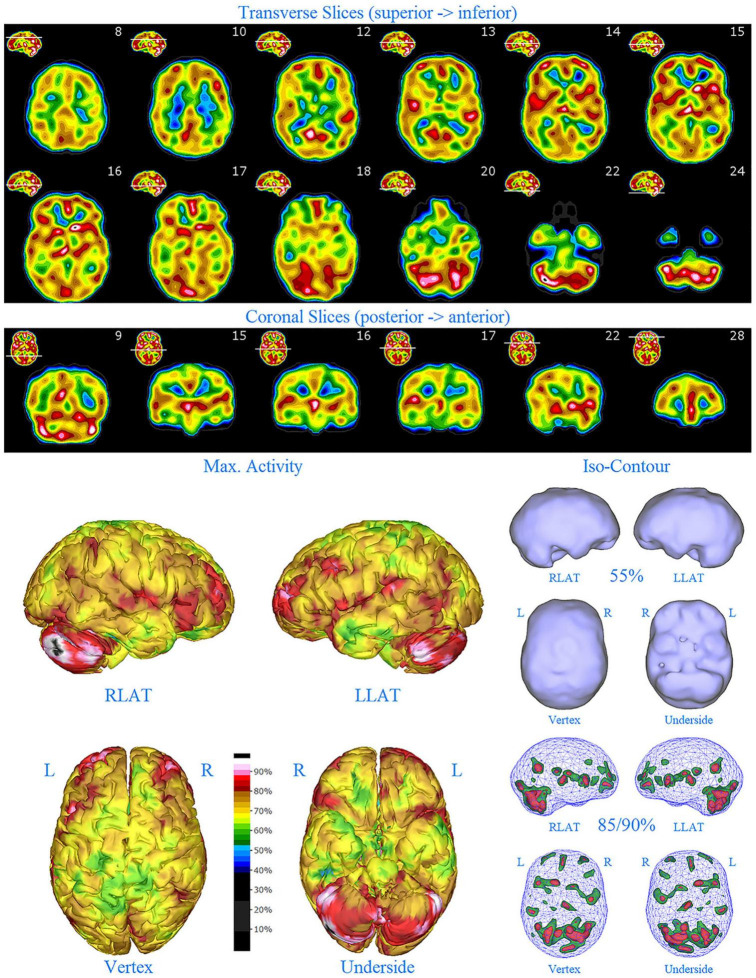
Perfusion SPECT scan of Patient B. Arrangement, color scales, and views are identical to Figure 1. Anatomical structures such as the thalamus are positioned similarly to the scan shown in Figure 2. Labeling was eliminated to avoid obscuring the scan details. Perfusion of the thalamus is increased and asymmetrical. Perfusion is markedly increased in the left caudate. Multiple cortical areas of increased perfusion are evident, predominately in the frontal cortices.

Despite her productivity, Patient B struggled with persistent anxiety, periodic outbursts of rageful anger, and low-grade depressive symptoms of low mood and guilt. The diagnostic picture was clouded. A SPECT Scan was ordered in 2012 (Figure 3). Based on the results of the SPECT scan, she was diagnosed with bipolar disorder and ADHD. Gabapentin was started and the dose of Adderall was increased. Patient B stabilized.

Unfortunately, the ongoing family psychiatric difficulties added to her stress. Gradually, all three of her children were put onto medication. Her daughter developed Bipolar Mixed State and ran away from home. However, her husband’s condition stabilized, and the family financial situation improved.

Because of depressive symptoms, Patient B started on Bupropion in 2014, without any benefit. At that point and based on the SPECT scan findings, Lamotrigine was added. After the dose was titrated to 100 mg per day, Lamotrigine provided relief. However, Patient B stopped both medications when she felt “back to her normal self.” She recently restarted Lamotrigine after discussion about ongoing potential benefits. She felt that a medication, in addition to Lisdexamfetamine, was necessary as she struggles with the stress of multiple family members who have physical and psychiatric illnesses.

SPECT scan findings for Patient B included: multiple cortical areas of increased perfusion (“hot spots”), increased perfusion of the posterior cingulate gyrus (right > left), increased perfusion in the cerebellum (right > left), and increased perfusion of the caudate head (left > right). Perfusion of the thalamus was also asymmetrical (right > left).

### Patient C

Patient C (the eldest child, a daughter) was stubborn and oppositional from a very young age. Nonetheless, she was able to follow a routine: at age 3 using an alarm clock, getting up, getting dressed, and eating breakfast, if left to do so independently. She was bright, a loner, and a voracious reader in the early years of school.

At age 9, Patient C was initially assessed psychiatrically for daydreaming and socially isolating, yet she was oppositional with adults at school. She noted “I don’t like school. I like reading. That is the only thing I like at school.” She was able to sit for a long time, but squirmed and wriggled. Often, she found school boring and did not like to listen. She noted that she didn’t like going to bed or getting up. Her creative play was very complex, and her self-organization a challenge. She met criteria for ADHD and ODD.

When started on Adderall, Patient C blossomed. Her parents found her pleasurable, rather than a drain on precious emotional resources. Unfortunately, early the next year, Patient C threatened to kill a child at after school daycare. The School Board reached out for an assessment. Notably, in total, she had three aggressive outbursts during her first year on stimulants.

**FIGURE 4:**
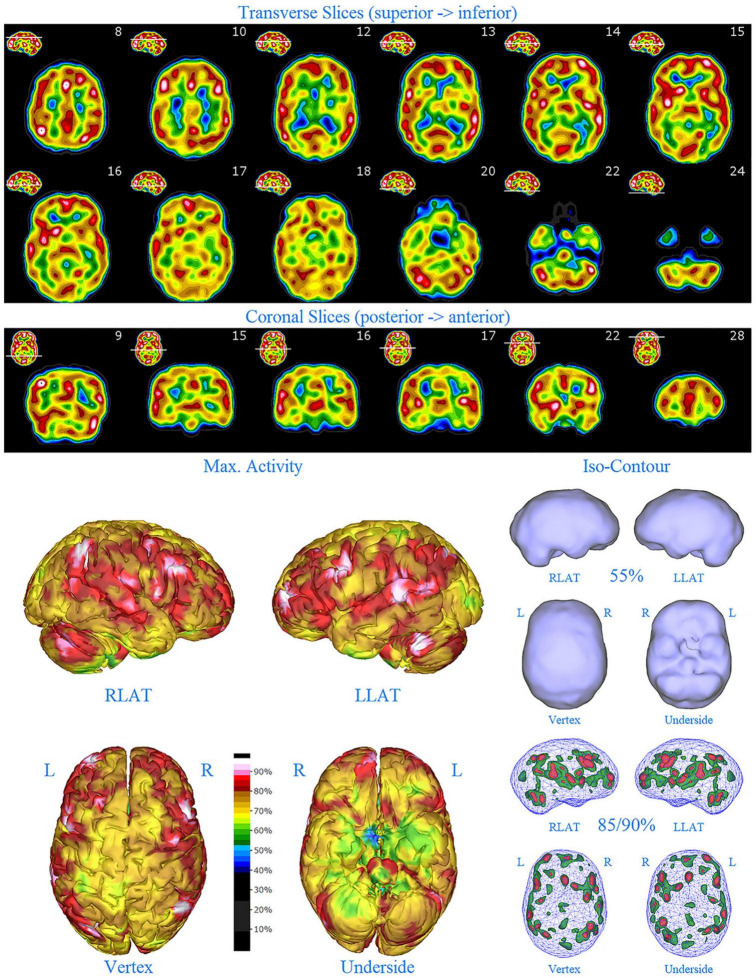
First perfusion SPECT scan of Patient C. Arrangement, color scales, and views are identical to Figure 1. Anatomical structures such as the thalamus are positioned similarly to the scan shown in Figure 2. Labeling was eliminated to avoid obscuring the scan details. Perfusion of the thalamus is essentially symmetrical. Perfusion is markedly increased in the right caudate. Multiple cortical areas of markedly increased perfusion are evident. For example, the highest signal is found in the right posterior parietal cortex (white area with black central dot in horizontal tomograms).

A SPECT scan was obtained at age 10 (2009) due to her extreme behaviors (Figure 4). Based on the SPECT scan results, a low dose of Gabapentin was added to Patient C’s regimen of Adderall to calm her aggression. Overall, Gabapentin proved helpful.

The following year, Patient C reported her parents to the Children’s Aid Society. It was unfounded and the investigator commented on how well the parents coped in a very difficult set of circumstances. A teacher commented at the time, that Patient C “is doing very well. She is a gifted writer and, as her parents point out, can embellish facts in her writing, and in her real life.”

Patient C, with her unusual and uncanny awareness of the family, commented “Mum is the hyper one, you’re (Father) the depressed one.” She commented to her youngest brother on their similarities “Although I explode ‘in’ you explode ‘out.”’ At the end of Grade 8, Patient C represented her age group on the Ontario School Council, a huge honor. She had a very positive summer working in a market stall and planting a large vegetable garden. She matured enormously and presented as a delightful young woman. She noted, “I could see I’m prone to Bipolar.”

**FIGURE 5:**
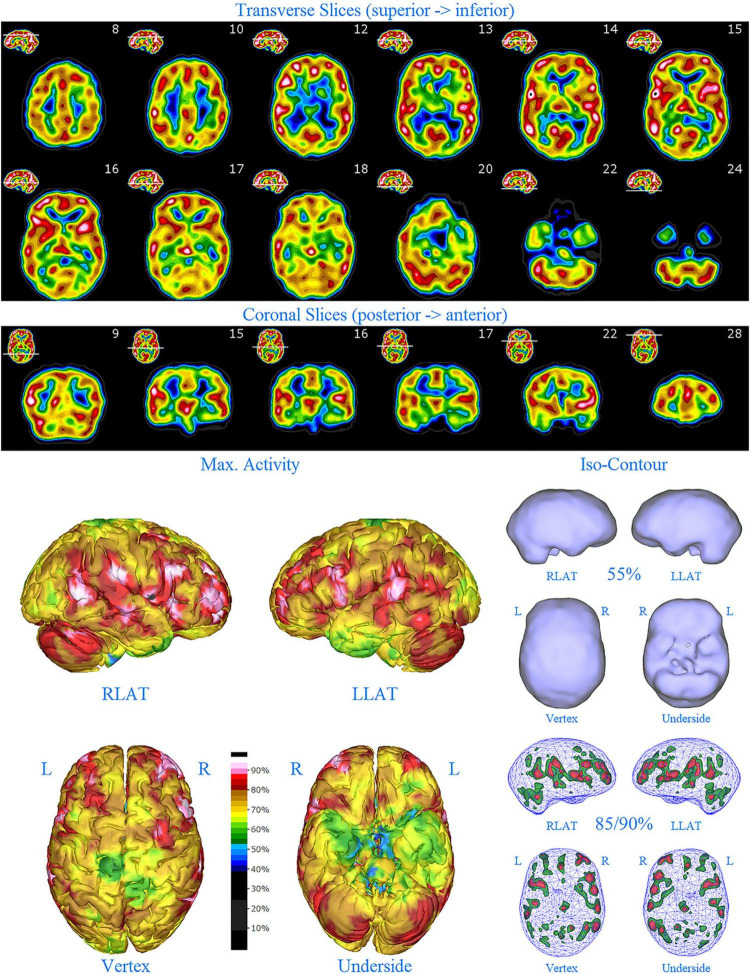
Second perfusion SPECT scan of Patient C. Arrangement, color scales, and views are identical to Figure 1. Anatomical structures such as the thalamus are positioned similarly to the scan shown in Figure 2. Labeling was eliminated to avoid obscuring the scan details. Perfusion of the thalamus is increased and asymmetrical. Multiple areas of markedly increased perfusion are found throughout the cerebral cortices. Also, areas of cortical hypoperfusion can be seen in the parietal cortices bilaterally (green on the 3-dimensional reconstruction).

Parents noted Patient C developed depressive symptoms in Grade 11. It was a challenging time in the family, with her father diagnosed with advanced cancer. Quite suddenly, as her final year of high school began Patient C became increasingly angry, irritable, anxious, and depressed. She also developed panic attacks. Her parents also were concerned about her increasing use of marijuana. A second SPECT scan (see Figure 5) was performed (2014).

Based on the findings in the second SPECT scan, Lurasidone and Clonazepam were begun, and Patient C expressed gratitude that she could be effectively treated. Her response to Lurasidone was initially favorable. Unfortunately, facts are difficult to ascertain as to the events of the subsequent 4 months. Patient C purportedly stopped taking Lurasidone and resumed taking Gabapentin and Lisdexamfetamine. Her mood worsened dramatically on a Christmas trip to see her grandparents. She became agitated and suicidal. In hindsight, marijuana withdrawal was a major factor.

A month later, when forbidden to smoke in bed, Patient C ran away from home. She completed her high school while living in a shelter for teenagers and working at a McDonald’s restaurant. She was estranged from her parents but had contact with her brother. He noted that she looked very anxious and could not make eye contact. Nevertheless, she then supported herself and saved for university.

Currently, Patient C is thriving academically at university, generally appreciative of family, but largely independent. She has moved from an interest in English and music to the sciences and hopes to pursue a pharmacy degree.

SPECT scan findings (Figure 4) for Patient C at age 10 (2009) included: multiple cortical areas of increased perfusion (“hot spots”), increased perfusion of the anterior cingulate gyrus (right > left), increased perfusion in the cerebellum (right > left), and increased perfusion of the basal ganglia (right > left). Notably, the perfusion of the thalamus was essentially symmetrical. The diffuse pattern of increased perfusion has been referred to as the “ring of fire,” indicative of pervasive over-activity in the cerebral cortex.

The second SPECT scan (see Figure 5) at age 16 again showed pervasive over-activity. In this scan, the perfusion of the thalamus is asymmetrical. Scattered areas of the parietal cortex showed significant hypoperfusion (appearing green on the 3-D image). This likely was due to extensive use of marijuana at that time.

### Patient D

Patient D (the second child, a son) is 2 years younger than his sister. He is, and was, the most easygoing of the three siblings. As a small child he was always very active physically. At home he was more oppositional, and easily slipped into a rage when roughhousing with friends. However, in school he was focused and productive. Reading was delayed.

Patient D met criteria for ADHD and Lisdexamfetamine was begun at age 9, with many benefits. Nonetheless, the parents saw many similarities between Patient D and Patient C in terms of oppositional behavior and sudden aggressive outbursts with peers. They requested a scan.

**FIGURE 6:**
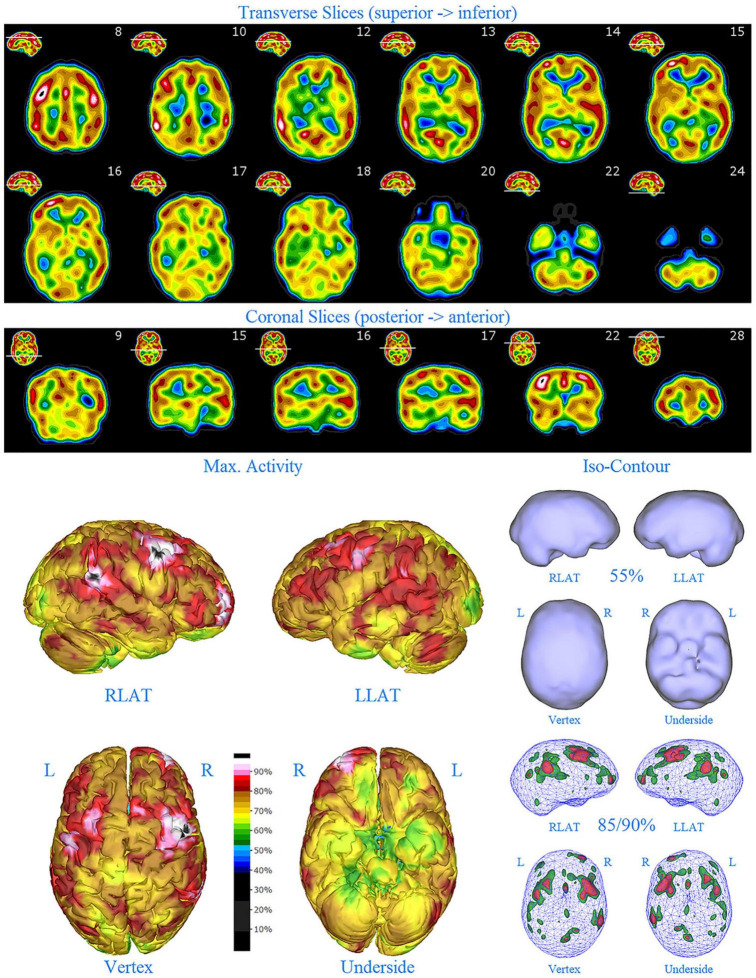
First perfusion SPECT scan of Patient D. Arrangement, color scales, and views are identical to Figure 1. Anatomical structures such as the thalamus are positioned similarly to the scan shown in Figure 2. Labeling was eliminated to avoid obscuring the scan details. Perfusion of the thalamus is increased and asymmetrical. Multiple cortical areas of markedly increased perfusion are evident in the right frontal and right parietal cortices.

The first SPECT scan for Patient D (Figure 6) was obtained at age 10 (2012). Based on the results of the scan, Gabapentin was added. Patient D responded favorably with reduced agitation, reduced oppositional behavior, fewer tantrums, and improved academic performance.

SPECT scan findings for Patient D included: multiple cortical areas of markedly increased perfusion (“hot spots”) in the right frontal and right parietal cortices, increased perfusion of the anterior cingulate gyrus, increased and asymmetrical perfusion of the thalamus, and normal perfusion of the basal ganglia (Figure 6). The diffuse pattern of increased perfusion has been referred to as the “ring of fire,” indicative of pervasive over-activity in the cerebral cortex.

**FIGURE 7:**
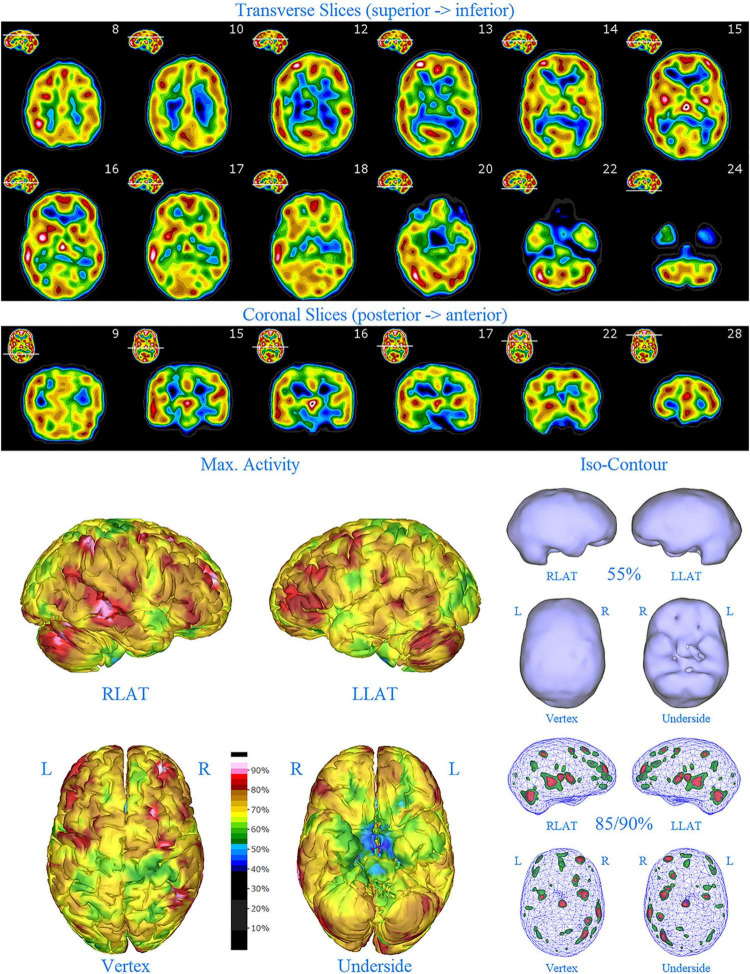
Second perfusion SPECT scan of Patient D. Arrangement, color scales, and views are identical to Figure 1. Anatomical structures such as the thalamus are positioned similarly to the scan shown in Figure 2. Labeling was eliminated to avoid obscuring the scan details. Perfusion of the thalamus is intensely increased and asymmetrical. Areas of increased perfusion in the cerebral cortices are considerably less prominent.

Patient D had several stable years. However, at age 13 he began to struggle with mood swings and other symptoms. He noted that he dreaded big assignments and did not tell anyone. “I fall off the horse and cannot get back on.” At times he had tons of energy and work was easy. At other times, he was lethargic, unmotivated, and overwhelmed. Regardless of whether he was “high” or “low,” he was more irritable, angry and non-compliant. A second SPECT scan (2015) was done due to the cyclical mood symptoms (Figure 7). The scan results revealed considerably less cortical overactivity (areas of increased perfusion), but the thalamus remained intensely perfused and asymmetrical. A plan was made to add a mood stabilizer, but it did not happen at the time because the family struggled with bigger issues.

In his first year of university during the COVID19 pandemic, Patient D became aware that he was struggling again and reached out for help. He remained on Lisdexamfetamine. He reported feeling he had been depressed most of his life but recalled the occasional high. He felt that working for a welder one day a week helped him through starting university in the COVID19 era. Patient D agreed to starting Lurasidone and demonstrated mood stabilization and improved academic success.

### Patient E

Patient E (the third child and younger son) was conceived at a time of challenges and financial instability within the family. By 2 years of age, he was called the “little emperor” due to the violent tantrums he would throw if he did not get his own way. For example, he vomited if his parents were upset with him. This terrorized his older brother, but his sister did not tolerate it. His family described him as an experiential learner. For example, at age 5, he dove from a tree house to see what it felt like to land face-first on the rocks 4 m below. Such impulsive behaviors – despite knowing that such behaviors were strictly forbidden led to many frightening episodes followed by deep remorse characterized by inconsolable crying and vomiting.

Patient E was first assessed psychiatrically at age 6 because of his odd behaviors, including hyperactivity, oppositionality, making frequent clicking sounds, screaming loudly, screeching, aggression with family members, and insomnia. He was diagnosed with ADHD and started on Lisdexamfetamine.

On his first day on Lisdexamfetamine, Patient E reported “lots of love inside.” He was less angry. The temper tantrums remained but were shorter and seemingly more intense (e.g., “I will kill you!”). However, he also experienced decreased appetite and bedtime became extended and more difficult with worsening insomnia. He also continued to demonstrate striking impulsivity, as described above. A low dose of Gabapentin was added and seemed to be somewhat helpful, but his insomnia remained.

**FIGURE 8:**
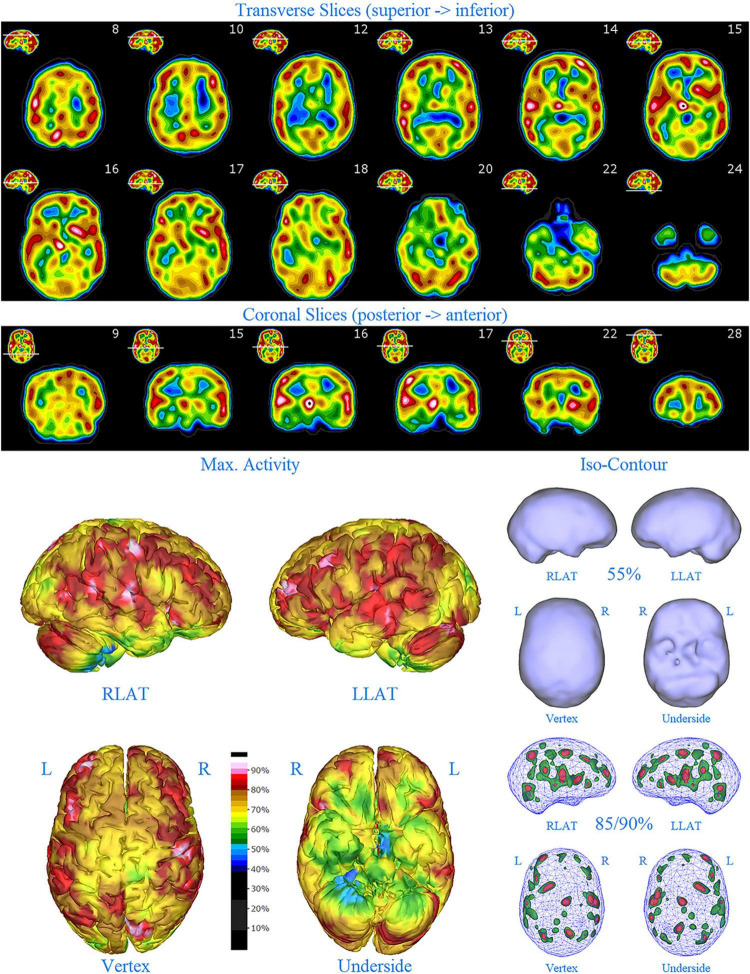
First perfusion SPECT scan of Patient E. Arrangement, color scales, and views are identical to Figure 1. Anatomical structures such as the thalamus are positioned similarly to the scan shown in Figure 2. Labeling was eliminated to avoid obscuring the scan details. Perfusion of the thalamus is intensely increased and asymmetrical. Multiple cortical areas of increased perfusion are seen throughout the cerebral cortices.

A SPECT scan was obtained on Patient E at age 6 (2012) to parse out if incipient bipolar disorder might be responsible for the partial response to Lisdexamfetamine and the continued mood symptoms (Figure 8). Based on the results of the scan, Risperidone 0.5 mg was started. The Risperidone provided immense relief. Patient E became happier, less anxious and more independent. He remained stable for several years.

SPECT scan findings (Figure 8) for Patient E included: multiple cortical areas of increased perfusion (“hot spots”) diffusely throughout the cortices, mildly increased perfusion of the anterior cingulate gyrus, increased and highly asymmetrical perfusion of the thalamus, and increased perfusion of the left basal ganglia. The diffuse pattern of increased perfusion has been referred to as the “ring of fire,” indicative of pervasive over-activity in the cerebral cortex.

**FIGURE 9:**
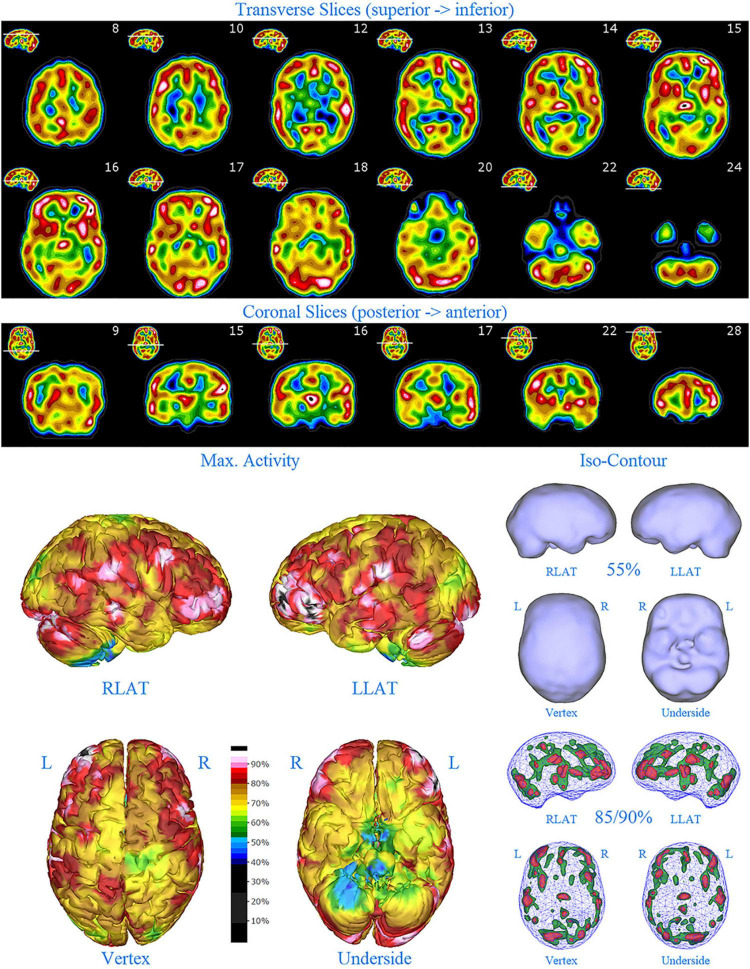
Second perfusion SPECT scan of Patient E. Arrangement, color scales, and views are identical to Figure 1. Anatomical structures such as the thalamus are positioned similarly to the scan shown in Figure 2. Labeling was eliminated to avoid obscuring the scan details. Perfusion of the thalamus is increased and asymmetrical. Areas of increased perfusion are considerably more prominent compared to the first scan, particularly in the left frontal cortex.

At age 9, Patient E began having multiple violent episodes of screaming, yelling, and physical violence toward his father. He also began endorsing impulsive suicidal thoughts. He endorsed racing thoughts and insomnia. Because of these rapidly escalating symptoms, a second SPECT scan was ordered on Patient E (2015). There was little change in the appearance of his two SPECT scans. Both showed asymmetric thalamic perfusion (more pronounced in the first scan) and diffuse increased cerebral perfusion, which had become even more pronounced at the time of the second scan. Based on the second SPECT scan findings (Figure 9), his dosage of Risperidone was increased, and Gabapentin was added. The patient responded favorably with significant modulation of his disruptive and unpleasant symptoms.

### *Post-hoc* Analysis

**FIGURE 10:**
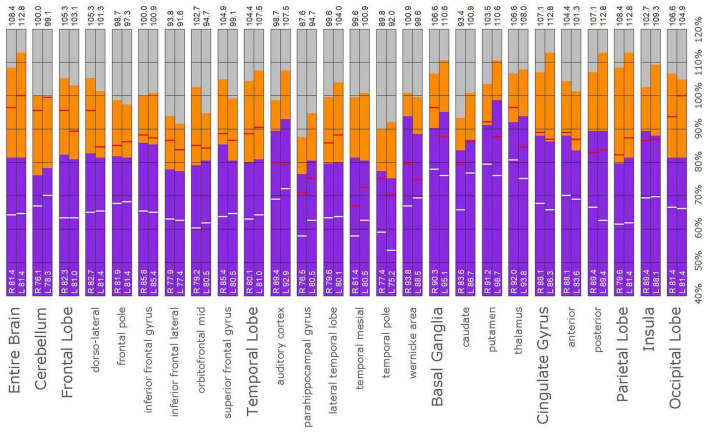
Semi-quantitative region of interest analysis of the SPECT scan of Patient A in comparison to the normative sample. The scan data was normalized to the cerebellar maximal counts (cerebellar maximal for each patient equals 100% on bar graph). The median counts in each ROI is shown in purple and the maximal counts in each ROI is shown in orange. Right and left regions are illustrated as separate bar graphs with the median value printed in white lettering at the base of each bar. All 67 regions could not be illustrated in a single graph, so multiple ROI’s from the frontal, temporal, and cingulate cortices are illustrated, while the parietal, occipital, and insular cortices are each illustrated by a single bar. The median counts of the normative sample for that particular ROI are illustrated as a white line on each bar of the graph. The maximum counts of the normative sample for that particular ROI are illustrated as a red line on each bar of the graph. Markedly increased perfusion is evident in the thalamus (exceeding the maximal value in the cerebellum and the comparable maximal value of the normative sample). Markedly increased perfusion is seen in the frontal, temporal, parietal, insular, and cingulate cortices, again exceeding the maximal perfusion of the cerebellum and the comparable maximal value of the normative sample. In all ROI, with the exception of the cerebellum, the patient’s maximal counts exceed that of the normative sample (red line). In all ROIs, the patient’s median counts exceed that of the normative sample (white line). The asymmetry evident in the thalamus upon inspection of the scan is less obvious with ROI analysis. Asymmetry is notable throughout the cortices.

**FIGURE 11:**
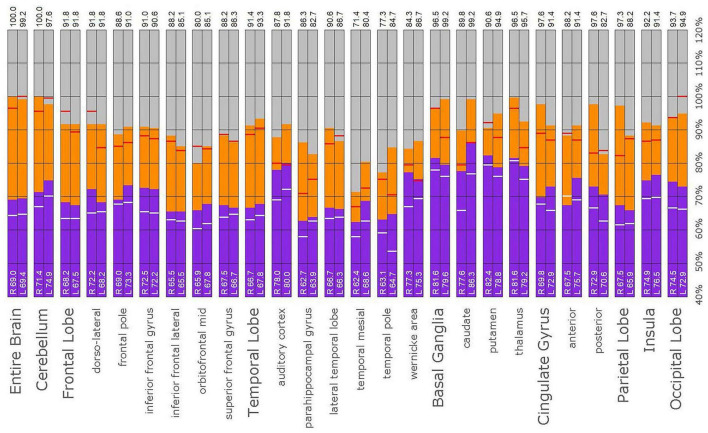
Semi-quantitative region of interest analysis of the SPECT scan of Patient B. Data analysis and presentation is as described in Figure 10. Perfusion of the thalamus is increased and asymmetrical, although not as obviously as upon inspection of the scan. Perfusion is markedly increased in the left caudate, approaching the maximal count of the cerebellum. Multiple cortical areas of increased perfusion are evident, predominately in the frontal cortices. In many ROIs, the patient’s maximal counts exceed that of the normative sample, though not to the degree seen in the other cases. Portions of the frontal cortex, the lateral temporal lobe, cingulate gyri, the putamen, and the occipital lobe, which show maximal counts less than that of the normative sample, are the exceptions. In all ROIs, the patient’s maximal counts exceed that of the normative sample (red line). In all ROI, the patient’s median counts exceed that of the normative sample, with the exception of the right anterior cingulate gyrus (white line).

**FIGURE 12:**
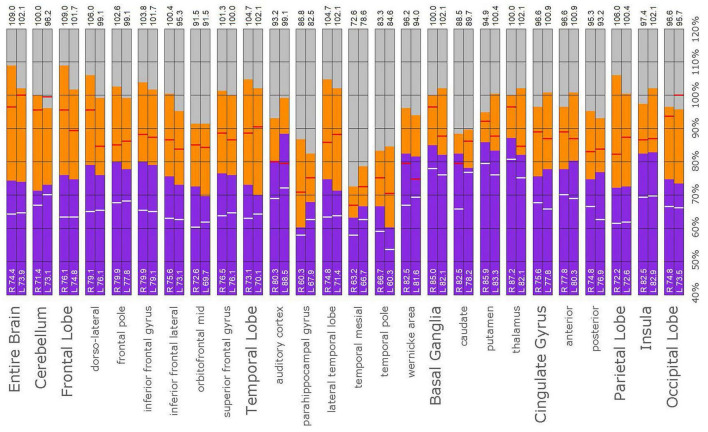
Semi-quantitative region of interest analysis of the SPECT scan of Patient C. Data analysis and presentation is as described in Figure 10. Thalamic perfusion is asymmetrical and exceeds that of the cerebellar maximum. Multiple cortical areas show increased perfusion which exceeds the cerebellar maximum and the comparable maximal value of the normative sample. Asymmetric perfusion is seen in most cortical areas. In all ROI, with the exception of the cerebellum and left occipital lobe, the patient’s maximal counts exceed that of the normative sample (red line). In all ROIs, the patient’s median counts exceed that of the normative sample (white line).

**FIGURE 13:**
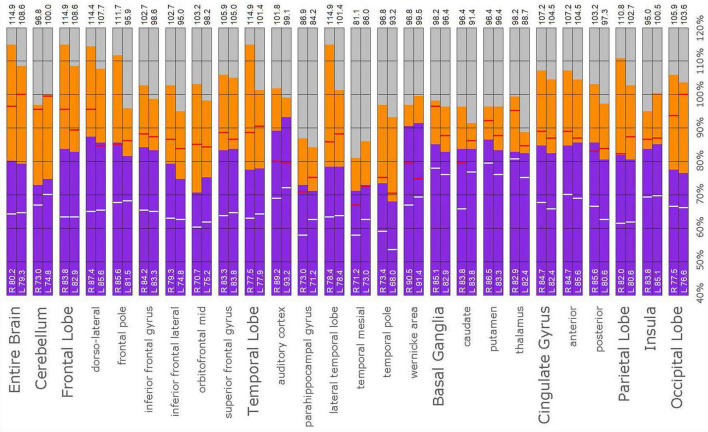
Semi-quantitative region of interest analysis of the SPECT scan of Patient D. Data analysis and presentation is as described in Figure 10. Thalamic perfusion exceeds the maximum in the cerebellum and is highly asymmetrical. The median and maximal counts in the thalamus exceed those of the normative sample. Multiple cortical areas of markedly increased perfusion are noted with marked asymmetry. In all ROI, with the exception of the cerebellum, the patient’s maximal counts exceed that of the normative sample (red line). In all ROIs, the patient’s median counts exceed that of the normative sample (white line).

**FIGURE 14:**
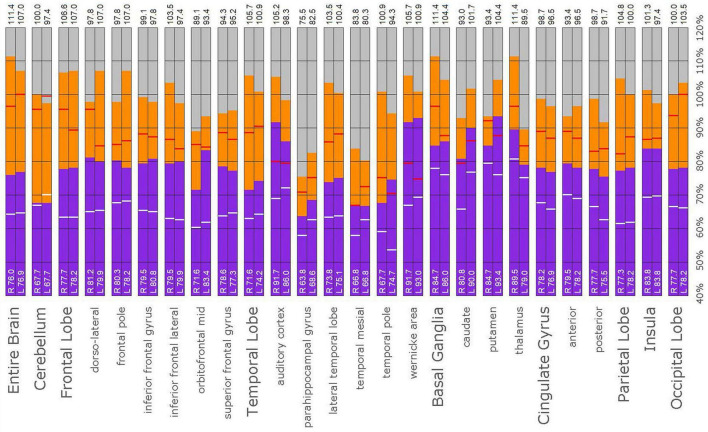
Semi-quantitative region of interest analysis of the SPECT scan of Patient E. Data analysis and presentation is as described in Figure 10. Thalamic perfusion exceeds the cerebellar maximum with marked asymmetry. Multiple cortical areas show asymmetrical and highly elevated perfusion exceeding the maximum in the cerebellum. Perfusion in the caudates and putamina is also elevated. In all ROI, with the exception of the cerebellum, the patient’s maximal counts exceed that of the normative sample (red line). In all ROIs, with the exception of the cerebellum, the patient’s median counts exceed that of the normative sample (white line).

The *post hoc* semi-quantitative analysis of the SPECT scans independently supported and validated the findings of the consensus opinion of the expert committee. Asymmetric perfusion was evident in multiple regions of the cerebral cortex, particularly the frontal and temporal lobes. Perfusion of the thalamus was asymmetrical in every case with the right thalamus showing higher counts in 3 out of the five cases. In addition, semi-quantitative analysis revealed that the right putamen had elevated perfusion frequently. Figures 10–14 show a bar graph representation for each patient. For each patient, the scan data was normalized to the cerebellum. Data for each ROI is represented as a bar graph. Most areas have two bars representing the left and right ROI. Some midline ROI are represented by a single bar graph, because the two areas are too close together to separate accurately. The orange bar graph represents maximal counts (perfusion) in the specific ROI and the purple bar represents mean count in the specific ROI relative to the specific patient in the specific scan. The median count of each ROI from the normative sample is represented by a white line. The red line illustrates the cerebellar maximal value for each ROI from the normative sample. Notably, in all cases cortical perfusion in at least one area exceeded the maximal perfusion in the cerebellum, which is usually the most highly perfused portion of the human brain. The findings illustrated in the semi-quantitative analysis match the description of findings in each case. For example, the scan of Patient B, the mildest of the cases clinically, showed frontal cortical areas approaching but not exceeding the cerebellar maximum (Figure 11). The thalamus is slightly asymmetrical and approaches the cerebellar maximum, but the left caudate is as highly perfused as the cerebellar maximum. Meanwhile, the second scan of Patient E showed multiple cortical areas of increased perfusion (“hot spots”) diffusely throughout the cortices, increased and highly asymmetrical perfusion of the thalamus, and increased perfusion of the left basal ganglia. The corresponding bar graph (Figure 14) shows multiple areas of asymmetric perfusion in the frontal, temporal and parietal (not shown) cortices, as well as increased perfusion of the right thalamus and increased perfusion of the left caudate and putamen.

## Discussion

In this retrospective review of a family cohort collected under naturalistic circumstances in the routine clinical practice of one of the authors, we have found two distinct findings. The first is asymmetric increased perfusion of the thalamus, which replicates earlier neuroimaging findings, regardless of the modality (SPECT, PET, fMRI). The second is patchy and markedly increased perfusion throughout the cerebral cortices, which also was asymmetrical. Each of the family members displayed asymmetrical perfusion of the thalamus (Patient D to the least extent, while Patient E provided the most striking example). This finding was often accompanied by asymmetric cortical perfusion as shown in the vertex view of the 3-D reconstruction images (best seen in Patient A, C, and D). All family members demonstrated patchy increased perfusion of the cerebral cortices. Distinct focal areas of intense perfusion were indicated by red areas with white central regions. These can be referred to as “hotspots.” The focal areas which were the most highly perfused point in the brain were marked by a black focus within the white central region (see Patient D 3-D reconstruction and tomograms – Figures 6, 7, for example). These ***potential*** diagnostic markers, which the authors and expert panel members have observed clinically in hundreds – if not thousands – of patients are ***potential*** endophenotypic markers for bipolar disorder.

### Increased Asymmetric Thalamic Perfusion

The finding of asymmetrical increased perfusion of the thalamus is supported by the observations of Juckel et al. (50), as well as others (47, 69, 70). Similarly, fMRI studies (58, 59, 71) reveal increased and often asymmetrical perfusion of the thalamus in patients with bipolar disorder. Again, our clinical observation across many patients with bipolar disorder support this finding.

### Diffuse Increased Cortical Perfusion

Diffuse increased cortical perfusion is evidence of elevated cortical activity. Compared to a normal scan in which the visual cortex is the most active cortical area (see Figure 1), the scans in the present series are strikingly different. Areas throughout the cortex, including the frontal, temporal, and parietal cortices are extremely active, often exceeding the maximal activity in the cerebellum (see Figures 2–9 and the same data presented in semi-quantitative graphic form in Figures 10–14). Notably, some areas are so active as to be “hotspots,” resembling in many ways the appearance of seizure foci on SPECT scan. This correlation between bipolar disorder and seizure disorders may have a molecular root. The ankyrin 3 gene (*Ank3*) regulates neuronal circuit activity and abnormalities in the function of one of the products of *Ank3*, a voltage-gated sodium channel, leads to excessive firing in circuits which are involved in emotions, memory, and epilepsy (72). *Ank3* is a leading candidate gene in bipolar disorder. If, indeed, this SPECT perfusion finding is associated with excessive firing in circuits involved in emotions, then medications which block or modify voltage-gated sodium channels (e.g., Carbamazepine, Lamotrigine, Valproic acid) would be expected to be effective for the treatment of bipolar disorder. Similarly, medications which block or modify voltage-gated calcium channels (e.g., Gabapentin, Pregabalin) likely will modulate the symptoms of bipolar disorder.

All members of the family showed diffuse increased cortical perfusion. All members responded favorably to a greater or lesser degree to Gabapentin. In Patient D, the improvement of cortical increased perfusion can be seen by comparing Figures 6 and 7. While, he still had troublesome symptoms and showed asymmetric thalamic perfusion, the overactivity of the cerebral cortices was reduced. In contrast, Figures 8 and 9 illustrate Patient E, who was on a low dose of Gabapentin at the time of both scans; yet, still had diffuse increased cerebral perfusion, along with disruptive behaviors, volcanic temper tantrums, racing thoughts, and insomnia – classic mania/hypomania symptomatology. Notably, after an increase in the dosage of both Gabapentin and Risperidone, his difficulties resolved.

The implication here is not that bipolar disorder is a form of seizure disorder. Rather, it is that the two phenomena share common features, both at the molecular level and at the whole-organ neurophysiological level as seen with SPECT scans and other forms of functional neuroimaging. Areas of increased cortical perfusion or “hotspots” should be taken into consideration when evaluating a SPECT scan for evidence consistent with the diagnosis of bipolar disorder.

### Detecting Prodromal/Incipient Bipolar Disorder

The issue of detecting prodromal or incipient bipolar disorder remains a critically unmet need in psychiatry. The clinical presentation of a first depressive episode gives no clue or insight into whether the patient has unipolar depression or a first depressive episode of bipolar disorder. In this retrospective review of a single-family case series, the overactive cerebral cortices appeared to be a consistent warning sign of the potential risk for activation or frank mania/hypomania if any of these patients were given a traditional antidepressant. This conclusion is supported by the adverse reaction displayed by Patient A when administered paroxetine. Based on the results of the SPECT scans, the clinician elected to avoid using antidepressants in these five patients.

As exemplified by the first scans of Patients D and E (obtained at age 10 and 6, respectively), the aforementioned neuroimaging “markers” were already quite evident at a young age. Given the reality that the diagnosis of bipolar disorder is often delayed by 5–10 years, as described above, these perfusion SPECT markers could serve to expedite the diagnosis of bipolar disorder. To reiterate, the predictive value of a neuroimaging marker need not be perfect in the situation of bipolar disorder wherein the risks of using medications appropriate for that diagnosis are relatively small. In contrast, the risks of delaying the diagnosis and giving medications which exacerbate bipolar disorder are substantial. For example, Henderson and Hartman (19) showed that the ADHD medication, Atomoxetine, can precipitate mania. The use of stimulants can lead to rapid cycling and more severe course of illness (12, 14–18). Notably, as a young adult Patient C was assessed by a different psychiatrist, who dismissed the diagnosis of bipolar disorder due to the absence of a documented manic episode. Nevertheless, the mood state prior to her second SPECT scan was most likely a mixed state, she responded favorably to Lurasidone, and her scan results demonstrated both neuroimaging markers.

**FIGURE 15:**
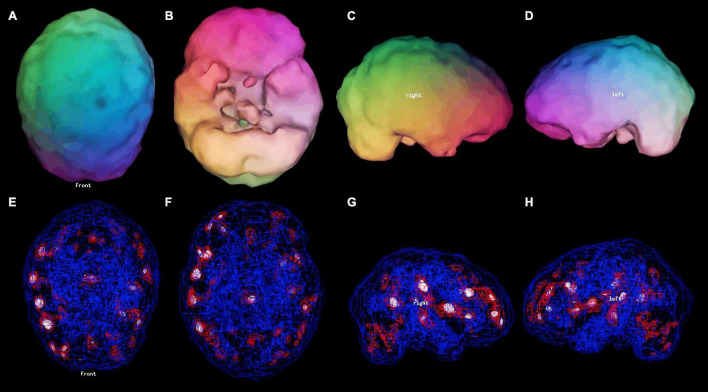
The second SPECT scan of Patient C shown as it was originally viewed after processing with the Picker Odyssey software. **(A–D)** 3-D representation of the scan data is shown. The surface is set at 60% of brain maximum. Areas which fall below 55% are represented as indentations or holes depending on how far below 55% the activity falls. **(A)** Vertex view, **(B)** Underside view, **(C)** Right Lateral, **(D)** Left Lateral. **(E–H)** A wireframe brain representation is shown, wherein the areas of brain with activity at 85% of maximum or greater are shown in red and areas of 92% or greater are shown in white. **(E)** Vertex view, **(F)** Underside view, **(G)** Right Lateral, **(H)** Left Lateral. The details of the SPECT scan are much harder to appreciate without the tomograms. The extent of the diffuse cortical increased perfusion is much more difficult to appreciate with this post-processing software. The extent of the co-existing areas of hypoperfusion in the parietal cortices are not evident with this post-processing software.

An additional observation can be made in this case series. Patient C demonstrated diffuse areas of hypoperfusion in her second scan, which is often associated with toxic brain injury. Later, it was learned that she was using marijuana heavily. Published clinical research (73, 74) and our extensive clinical experience has shown that the use of marijuana can lead to varying degrees of toxic encephalopathy. Indeed, decreased perfusion in the hippocampus can be closely correlated to reported frequency of marijuana use (74). On a related technical note, the relatively subtle hypoperfusion which signaled these neurotoxic changes were not appreciated on the decades-old processing software originally used to examine the scan of Patient C (Figure 15). Advances in SPECT scan camera technology and in post-processing software are yielding marked increases in the information which can be derived from perfusion SPECT scans (75).

While this case series is unique in that it consists of members of a single nuclear family, there are certain limitations. First, it represents a very small sample. Second, the data were interpreted without blinding to the clinical circumstances, as is typical for case series. Third, the patients were treated individually and in a naturalistic setting. Treatment was not uniform. As such, the present data can not define a biomarker. However, these data suggest certain neuroimaging findings may be reliable, providing initial evidence for a marker with reasonable uniformity in at least this small population. These findings warrant further investigations into perfusion SPECT neuroimaging biomarkers for a bipolar phenotype. These limitations notwithstanding, the use of perfusion SPECT scans has a place in the psychiatric evaluation of patients, as evidenced by the incorporation of psychiatric indications into the Canadian Association of Nuclear Medicine Guidelines for Brain Perfusion Single Photon Emission Computed Tomography (SPECT) published in 2021 and the recent comprehensive review of perfusion SPECT neuroimaging (76).

### Incorporating SPECT Scans Into Psychiatric Practice

#### SPECT Scans as a Tool

Perfusion SPECT scans have been vilified because they do not match up one-to-one with DSM-5 diagnoses (77). It is not surprising that SPECT scans do not yield a pathognomonic imaging result for each DSM condition. The diagnoses in the DSM-5 are replete with overlapping symptoms, comorbidity, and an absence of neurophysiological correlates (48, 78). Moreover, there is often tremendous range in how patients’ symptoms present. For example, using the DSM-5 (77) diagnostic criteria for Major Depressive Disorder (79, 80), there are over 20 possible distinct phenotypes of this single diagnosis. After multiple clinical trials failed to treat major depression, one expert stated,

*“that major depressive disorder is biologically heterogeneous, such that different treatments differ in the likelihood of achieving remission in different patients”* (81).

On the other hand, a common neurophysiological process, such as abnormally functioning voltage-gated channels due to *Ank-3* genetic anomalies, might lead to symptoms consistent with ADHD, ODD, intermittent explosive disorder, or disruptive mood dysregulation disorder. Furthermore, perfusion SPECT neuroimaging can help rule out toxic injury or traumatic brain injury–both of which can also lead to impulsivity, inattention, and mood symptoms (80).

In other words, perfusion SPECT scans are a tool. They help the physician understand the functioning of the brain better. SPECT findings might lead to an alternate differential diagnosis list. For example, an oppositional and defiant child with markedly increased cortical perfusion most likely has a different diagnosis than a similar behaving child who has significantly decreased cortical perfusion. Alternatively, as described by Pavel et al. (75), a patient can present with symptoms suggestive of bipolar mania, but have a diffusely hypoperfused brain suggesting infection, toxicity, or autoimmune disease. Follow-up testing in that particular case described by Pavel et al. (75) demonstrated infection, rather than bipolar disorder. The patient was correctly treated with antibiotics and a lifetime of psychotropic medications was averted. SPECT scans can aid clinicians to unravel complex cases.

#### Patient Confidence and Compliance

One barrier to the diagnosis of bipolar disorder is stigma. Patients are loath to accept the diagnosis and psychiatrists are reluctant to make the diagnosis (6). A very large threshold of evidence must be surmounted before the diagnosis can be made. From the perspective of psychiatrists among the authors, this diagnostic hesitancy is almost unforgivable in the modern era. It seems to be a holdover from the days when we only had lithium and electroconvulsive shock therapy to treat bipolar disorder. However, modern pharmaceuticals, such as lurasidone, cariprazine, lamotrigine, and others carry far fewer risks and much more manageable side effects. Moreover, most of them have antidepressant qualities which, in some cases, are superior to the serotonin reuptake inhibiting antidepressants. Nevertheless, the hesitancy exists.

For patients, perfusion SPECT brain scans bring a certain degree of confidence in their diagnosis. This was clearly evident in the family reported herein. The first member of the family to undergo brain SPECT scanning was the daughter (Patient C) due to her aggressive behavior. After hearing the description of the benefits and risks of a SPECT scan, the father (Patient A) requested to undergo a scan, as well. He expressed the desire to understand how his brain was working. Similarly, when both sons were scanned, the mother (Patient B) requested that she also be scanned. All family members have independently expressed that the scans helped them understand why they had the symptoms they had and how the selected medications could be beneficial. Both parents were very concerned that medications were used sparingly and as effectively as possible. They found the SPECT scans guided this process well.

The SPECT scans gave the family confidence that their treatments would be helpful. The scans also strengthened the compliance of each patient. For example, the younger son (Patient E) has been extremely compliant with a mood stabilizer from a young age. He notes that his medication greatly helps his sleep and his anxiety. Similarly, the older son (Patient D), who was actually inadequately treated during his high school years, reached out to the psychiatrist during his university years and self-identified symptoms of anxiety and hypomania. He requested to start taking a mood stabilizer.

Lastly, the presence of bipolar disorder, in and of itself, has been shown to increase the risk of suicide, up to 14-fold (28), underscoring the importance of early and correct diagnosis. Children with bipolar disorder are at higher risk for suicide than children with unipolar depression (82, 83). Suicide attempts are considerably more likely to be lethal among those with bipolar disorder (84) and occur at a younger age (83, 84). Recently, Orsolini et al. (85) thoroughly reviewed suicide and depressive disorders, noting the interwoven roles of neurobiological, neuroimmunological, and psychosocial factors. Recently, the COVID-19 pandemic has increased the factors that can contribute to suicidal ideation (86), as exemplified by Patient D. The multi-faceted etiological factors described elegantly by Orsolini et al. (85) underscore the value of a single-family study, such as this, wherein some of the variables can be controlled. Earlier diagnosis, patient belief and confidence in the diagnosis, increased insight, and enhanced therapeutic alliance, as exemplified in this case series, improve medication adherence (29) and reduce suicide risk (28, 84, 85).

#### Minding the “Hotspots”

Another important aspect of incorporating SPECT scans into psychiatric practice is that both areas of *underactivity* (as seen in depression, brain injury, stroke, ADHD, etc.) and areas of *overactivity* (as seen in bipolar disorder, obsessive-compulsive disorder, post-traumatic stress disorder, etc.) are important. The traditional training in Nuclear Medicine is to pay attention to the areas of decreased activity with decreased tracer uptake (hypoperfusion). This allows the nuclear medicine physician to identify brain injury, dementia, and strokes–the most common indications for which SPECT scans are ordered. In the psychiatric realm, clinicians must not only be mindful of these possible diagnoses, but also of the diagnoses that can lead to increased activity with increased tracer uptake (increased perfusion), such as illustrated in the present case series. Collaboration between the psychiatrist and the nuclear medicine physician become essential, along with displaying the scan data in such a manner, as illustrated herein, that “hotspots” can be identified. Notably, a color scale rather than a gray scale is needed to highlight areas of increased perfusion, as well as areas of diffuse hypoperfusion (in contrast to focal areas of hypoperfusion) which are much harder to appreciate in gray scale (75, 76). More importantly, a 3-D presentation of the data allows the focal areas of increased perfusion to be more prominent and more easily localized compared to just reading the scan in tomograms. In this case series, the “hotspots” in Patient B (Figure 3) and Patient C (Figure 4) were much harder to appreciate in tomograms.

These findings, along with recent publications (49, 76), point the way to future collaborations between nuclear medicine physicians and psychiatrists. Until recently, very few nuclear medicine physicians had experience with neuropsychiatric diagnoses, because nuclear medicine physicians receive few referrals for neuropsychiatric disorders and so have little clinical experience with patterns of increased perfusion. This situation is aggravated by the absence of referrals for neuropsychiatric evaluations, compounded recently by the American Psychiatric Association dismissing neuroimaging as a valuable adjunct in the evaluation of psychiatric patients (48, 78, 80, 87). Hopefully, enlightened clinicians in both fields will join together to deepen their clinical experience to advance and expand our understanding of the neuroimaging correlates in neuropsychiatry. As psychiatric and nuclear medicine practitioners, the authors and the International Society of Applied Neuroimaging have developed working models of how neuroimaging can contribute to the evaluation of, and more rapid diagnosis and successful treatment of, neuropsychiatric patients.

## Conclusion

We have presented a retrospective review of a family cohort including every member of a single family collected under naturalistic circumstances that illustrates clinical symptom development and parallel brain perfusion findings consistent with bipolar disorder. The advantage of this single-family study is that many socioeconomic, developmental, and social variables were controlled. As a result, and consistent with our extensive experience in interpreting SPECT scans, these perfusion findings have substantive correlation to bipolar disorder. These correlations *suggest potential* perfusion SPECT biomarkers for bipolar disorder. The potential biomarkers of: (1) asymmetric and increased perfusion of the thalamus and (2) diffusely increased cortical perfusion (that may be asymmetrical) proposed herein warrant further attention. The increased cortical perfusion or “hotspots” facilitates the differentiation of bipolar depression from unipolar depression, which tends to show symmetrical increased perfusion in the thalamus and basal ganglia, but hypoperfusion throughout the frontal and temporal cortices. Recognizing that bipolar disorder is increasingly seen as a spectrum of symptoms, rather than the simplistic bipolar I and II dichotomy, symptoms such as mania, hypomania, smoldering mixed mood episodes, impulsivity, irritability, and anxiety can all exist together or separately at various times in the life of a patient with bipolar disorder. Hence, a symptom-based diagnostic strategy would benefit greatly from neuroimaging correlates or biomarkers. These data encourage further study of larger populations with comparison to normative databases and/or control groups to test the robustness of these potential biomarkers for bipolar disorder.

More general and widespread awareness of the information provided by SPECT scans would likely improve: (1) the latency to diagnosis, (2) treatment, and (3) lifetime outcome of patients suffering from bipolar disorder. Patient confidence in their diagnoses would likely improve, leading to increased treatment compliance. Improved display parameters, as presented herein, facilitate the interpretation of SPECT scan data, allowing for wider acceptance and adoption of SPECT scans as a tool to aid in the diagnostic process.

## Data Availability Statement

The raw data supporting the conclusions of this article will be made available by the authors, without undue reservation.

## Ethics Statement

Ethical review and approval was not required for the study on human participants in accordance with the local legislation and institutional requirements. Written informed consent to participate in this study was provided by the participants’ legal guardian/next of kin. Written informed consent was obtained from the individual(s), and minor(s)’ legal guardian/next of kin, for the publication of any potentially identifiable images or data included in this article.

## Author Contributions

MM was responsible for care of patients, analysis, and writing of manuscript. TH involved in analysis, figure preparation, and writing of manuscript. DP involved in analysis of data and preparation of preliminary drafts of manuscript. PC involved in analysis of data and editing of manuscript. All authors contributed to the article and approved the submitted version.

## Conflict of Interest

TH is the president and principal owner of The Synaptic Space, a neuroimaging consulting firm. He is also CEO and Chairman of the Board of Neuro-Luminance Corporation, a medical service company. He is also president and principal owner of Dr. TH, Inc., a medical service company. He is also Vice-President of the Neuro-Laser Foundation, a non-profit organization. He is a member of and a former officer of the Brain Imaging Council Board of the Society of Nuclear Medicine and Molecular Imaging (SNMMI). Since 2017, he has served in the SNMMI Brain Imaging Outreach Working Group. Currently, he serves as president of the International Society of Applied Neuroimaging. TH has no ownership in, and receives no remuneration from, any neuroimaging company. No more than 5% of his income is derived from neuroimaging. DP was Director of PathFinder Brain SPECT which is a clinical service corporation providing SPECT functional neuroimaging and had no research funding. The remaining author declares that the research was conducted in the absence of any commercial or financial relationships that could be construed as a potential conflict of interest.

## Publisher’s Note

All claims expressed in this article are solely those of the authors and do not necessarily represent those of their affiliated organizations, or those of the publisher, the editors and the reviewers. Any product that may be evaluated in this article, or claim that may be made by its manufacturer, is not guaranteed or endorsed by the publisher.
